# Clinical Audit on Badminton-Related Ocular Injuries in a Tertiary Hospital in Malaysia

**DOI:** 10.7759/cureus.30769

**Published:** 2022-10-27

**Authors:** Abd Hadi Mohd Rasidin, Mohamad Kamil Muhammad-Ikmal, Raja Norliza Raja Omar, Azhany Yaakub, Liza Sharmini Ahmad Tajudin

**Affiliations:** 1 Department of Ophthalmology and Visual Science, School of Medical Sciences, Universiti Sains Malaysia, Kubang Kerian, MYS; 2 Department of Ophthalmology, Hospital Melaka, Melaka, MYS; 3 Ophthalmology Clinic, Hospital Universiti Sains Malaysia, Kubang Kerian, MYS; 4 Department of Ophthalmology, Faculty of Medicine and Health Sciences, Universiti Malaysia Sabah, Sabah, MYS

**Keywords:** ocular sports injuries, shuttlecock injuries, badminton-related ocular injuries, angle recession glaucoma, traumatic hyphema

## Abstract

Background

Badminton-related ocular injuries are among the commonest causes of blunt trauma to the eye, which can lead to significant damage to the ocular structures. This study aimed to assess the clinical presentations, complications, and visual outcomes of patients who sustained ocular injuries related to badminton treated in a single tertiary center in Malaysia.

Materials and methods

A retrospective clinical audit was conducted in Hospital Universiti Sains Malaysia (HUSM), Malaysia, involving patients diagnosed with ocular injuries related to badminton, either as players or spectators, between January 1, 2003 and December 31, 2017. The demographic data, mechanism of injury, and clinical presentation were recorded. In addition, visual acuity, anterior and posterior segment, and intraocular pressure (IOP) measurements were recorded at the initial presentation and at the present recruitment period. Management at the initial presentation was also obtained and recorded. The final visual outcome and complications were based on the finding of the most recent follow-up. Visual acuity was categorized as follows: mild or no visual impairment (6/18 or better), moderate and severe visual impairment (<6/18 and worse).

Results

A total of 23 patients (23 eyes) were included in this clinical audit. The average age was 24 years, with a range of 6-56 years, with the highest incidence occurring at the age of 20 years old and younger. The majority of the injuries were sustained during the single-player game. All the injuries were caused by shuttlecock hits. In 18 cases (78%), the trauma was caused by an opponent, in four cases (17%) by a partner, and in one case involving a bystander. Most of the patients in this series were not using any protective eyewear while playing the game 96% (22). Most injuries (22 eyes) involved the anterior segment, with hyphaema as the commonest clinical presentation. The mean IOP at presentation was 23.5 (11.2) mmHg. Angle recession was detected as early as one-week post initial presentation in 17 eyes. Commotio retinae (5 eyes) and vitreous hemorrhage (4 eyes) were the common posterior segment findings. There were eight eyes with visual acuity of worse than 6/18 at the initial presentation, but only three eyes had poor final visual acuity. There was a statistically significant improvement in visual acuity at the last follow-up compared to the initial presentation (Fisher’s exact test) (p=0.032).

Conclusion

Ocular injuries related to badminton is common and cause a detrimental effect on the long-term visual outcome. Traumatic hyphaema and commotio retinae are the most common presenting signs related to poor visual outcomes. Therefore, protective eyewear and promoting awareness of badminton-related ocular injuries are essential to prevent monocular blindness in young adults.

## Introduction

Ocular trauma is the leading cause of monocular blindness, with an annual incidence of 55 million cases, and 750,000 require hospitalization [1,2]. There are various causes of ocular trauma, including sports-related injuries. Sport-related injury contributed to 25-40% of total admission of ocular trauma [3]. It is less common compared to sport-related musculoskeletal injuries. Nonetheless, it is responsible for significant morbidity and function loss, especially among the reproductive age group [4]. Naturally, due to their risk-taker behavior, young men are primarily involved in sport-related ocular trauma [5]. Sport-related ocular trauma is closely related to the popular type of sport in the region or country. Badminton is one of Asia's most popular sports, involving a single player or two players. Shuttlecock smashed by the opponent in a single game or hit by the racket of a partner in a double game cause direct injury to the eye [6]. A shuttlecock is smaller in size compared to a tennis ball. There is a hard material at the base and feathers for trajectory effect [7]. The eye is an enclosed round structure protected by corneal and sclera. The high-velocity projectile trajectory of a shuttlecock may cause blunt or even penetrating injury to the eye [8]. Blunt trauma causes sudden compression and decompression at the equator of the eye, which may result in hyphaema, traumatic cataract, cyclodialysis, iridodialysis, sphincter tears, subluxation of the lens, vitreous hemorrhage, and Berlin's edema [8]. There are also reported late-onset complications such as cystoid macular edema, retinal detachment, traumatic optic neuropathy, pigmentary changes in the macula, and angle recession glaucoma [8]. Badminton-related injury is responsible for 64.4% of hyphema secondary to blunt trauma [6]. In the United Kingdom, badminton-related injuries are responsible for 19% of severe sports-related eye injuries [8]. While in Canada, badminton-related eye injuries have been rising since 1976 and are severe enough to cause blindness [7,8]. The majority of the injuries are caused by the shuttlecock [9]. In Malaysia, badminton-related injuries are responsible for two-thirds of all sport-related ocular trauma [10]. Therefore, understanding the clinical presentation and long-term outcome of ocular injury related to badminton is essential in the prevention of blindness in Malaysia. This study was conducted to determine the clinical manifestation, complications, and visual outcome of patients who sustained badminton-related ocular injuries treated in a single tertiary center in Malaysia.

## Materials and methods

This clinical audit was conducted through a retrospective record review in Hospital Universiti Sains Malaysia (HUSM), Malaysia, involving patients diagnosed with ocular injuries related to badminton either as a player or spectator between January 1, 2003 and December 31, 2017. The definition of ocular injuries is adopted from Birmingham Eye Trauma Terminology (BETT) [11]. In this study, only closed-globe injuries were included. Closed-globe injury is defined as any injury sustained during a badminton game that causes partial or no discontinuation of the eye wall. All recruited patients must complete at least a year of follow-up between January 1, 2004 and December 31, 2018. This study received ethical approval from the Human Research Ethics Committee of USM (JEPeM) and is conducted in accordance with the declaration of Helsinki for human research.

Medical record of patients diagnosed with ocular trauma was traced from the electronic database. Only those records that provide clear evidence of ocular injuries sustained while playing or watching badminton (bystander) games were included. Ocular injuries must be due to direct or indirect hits by the shuttlecock or racket during recreational or sports tournaments. Any injuries due to falls or slips during the game were excluded. Those with unclear history of direct injury related to badminton were excluded. They were also excluded if there was more than 30% of missing data from the medical record.

The demographic details, including age, gender, ethnicity, and details about the ocular injury, including date of injury and affected eye, were entered into a proforma. The age group was stratified into 20-year intervals. The following sports-related data were collected: type of games (singles or doubles), instigator of the injury (partner or opponent), the instrument of the injury (shuttlecock or racquet), and use of protective eyewear (yes or no). Ophthalmological data were retrospectively extracted from the medical records, including visual acuity, correction of refractive error, anterior and posterior segment findings, and intraocular pressure (IOP) measurement at the initial presentation and the present recruitment period. The type of injury and management at the initial presentation, including the admission or outpatient treatment, were also obtained and recorded. Severe hyphaema and poor visual acuity at presentation are among the reason that warrants hospital admission. The final visual outcome and complications were based on the finding of the most recent follow-up. Visual acuity was categorized as follows: mild or no visual impairment (6/18 or better) and moderate and severe visual impairment (<6/18 and worse).

All data were entered into the SPSS version 26.0 software (IBM Corp, Armonk, NY). They were checked to ensure accurate documentation and eliminate any missing or erroneous values. A comparison between visual acuity at presentation and final follow-up was made using Fisher's exact test. P-value <0.05 was deemed statistically significant in this study.

## Results

This clinical audit involved 23 eyes from 23 patients, with 22 (96%) of them admitted for moderate and severe injuries. The mean duration of follow-up was two years. The majority were men. The average age was 24 years, with a range of 6-56 years, with the highest incidence between 0 and 20 years old (Table 1). Five patients had a pre-existing refractive error, with four being myopic and one hyperopic.

**Table 1:** Demographic data.

Variables		n (%)
Race	Malay	21 (91.0)
	Chinese	2 (9.0)
Age (year)	0-20	12 (52.0)
	21-40	5 (22.0)
	41-60	6 (26.0)
	Mean	24
	Median	17
Sex (n, %)	Male	22 (96.0)
	Female	1 (4.0)
Refractive error	Emmetropia	18 (78.3)
	Myopia	4 (17.4)
	Hyperopia	1 (4.3)
Admission	Inpatient	22 (96.0)
	Outpatient	1 (4.0)
Follow up duration (months)	Range	6-120
	Median	12

The mechanism of injury is shown in Table 2. In our series, the majority of the injuries were sustained during single-player games. Shuttlecock from the opponent in a single game was responsible for 78% (18) of injuries and 17% (4) due to their own partner's shuttlecock in a double game, and one patient was hit by the shuttlecock while watching the game (bystander). There was no recorded ocular injury sustained by a badminton racket in this study. The majority of the patients, 96% (22), in this series were not using any protective eyewear while playing the game.

**Table 2:** Mechanism of injury.

Variables		n (%)
Type of game	Single	19 (83.0)
	Double	4 (17.0)
Type of injury	Shuttlecock	23 (100.0)
	-Opponent	18 (78.3)
	-Partner	4 (17.4)
	-Hit a bystander	1 (4.3)
	Racket	0 (0)
Wearing eye protection	Yes	1 (4.0)
	No	22 (96.0)

The majority of the injuries (22 eyes) involved the anterior segment, with hyphema as the commonest clinical presentation (Table 3). The mean IOP at presentation was 23.5 (11.2) mmHg. Angle recession was detected as early as one-week post initial presentation in 17 eyes. In comparison, commotio retinae (5 eyes) and vitreous hemorrhage (4 eyes) were the common posterior segment findings. The severity of the impact has resulted in a patient presenting with a retinal tear and four patients with subluxated lenses (Table 3).

**Table 3:** Ocular manifestation at presentation post badminton-related ocular trauma. IOP: Intraocular pressure; PVD: Posterior vitreous detachment.

Ocular findings		
IOP (mmHg)	Mean (SD)	23.5 (11.2)
	Range	10-49
Anterior segment (n(%))	Corneal abrasion	3 (13.0)
	Iridodialysis	1 (4.0)
	Hyphema	22 (96.0)
	Angle recession	17 (74.0)
	Lens subluxation	4 (17.0)
Posterior segment (n(%))	PVD	1 (4.0)
	Vitreous haemorrhage	4 (17.0)
	Retinal tear	1 (4.0)
	Commotio retinae	5 (22.0)

**Figure 1:**
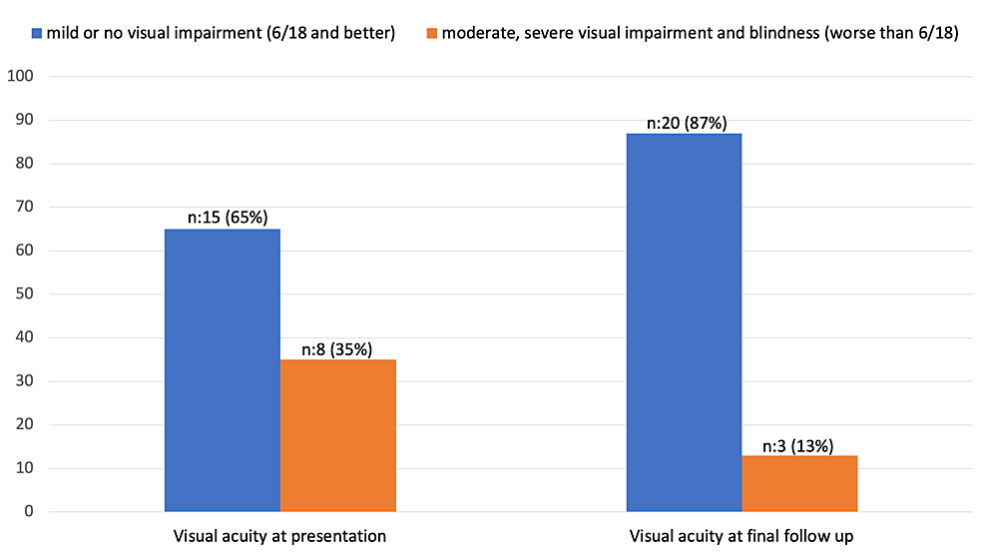
Visual acuity at initial presentation and final follow-up. p=0.032 based on Fisher's exact test.

There were eight eyes with visual acuity worse than 6/18 at the initial presentation, but only three had poor final visual acuity. There was a statistically significant improvement in visual acuity at the last follow-up compared to the initial presentation (p=0.032) (Figure 1). Those patients with traumatic cataracts secondary to badminton-related injuries all had their lenses removed at the time of the recruitment period of this study. Most of them (74%) developed traumatic angle recession, with eight patients developing elevated IOP but without evidence of glaucomatous changes. While four already showed structural and functional damage to glaucoma (Table 4).

**Table 4:** Ocular complications. IOP: Intraocular pressure.

Ocular findings	n (%)
Traumatic angle recession	17 (74.0)
-Gonioscopic changes	17 (100.0)
-Elevated IOP	8 (47.0)
-Developed glaucoma	4 (24.0)
Traumatic cataract	8 (35.0)
Retina-related complication	3 (13.0)

## Discussion

Badminton is considered a national sport in many countries, especially in Asia. Thus, badminton is a popular recreational sport played by all age groups [12]. It is known that badminton is responsible for more severe injuries compared to other sport-related injuries [13]. A population-based study conducted in Denmark found that the commonest injury was muscle sprain and a small number of ocular injuries [14]. Perhaps, due to a good health system, the impact of badminton-related injury in Denmark is not as devastating as reported in other countries [15]. In the present study, we did not include other types of badminton-related injuries except ocular ones.

Badminton involves multiple types of shots, such as the smash, the clear, the drop, the net shot, and the drive. Smash shot involves the high velocity of a flying shuttlecock that can reach the speed of 400 km/h [16]. Similarly, in this study, all injuries were due to flying shuttlecock either from the opponent or partner in a double game. The hard base of the shuttlecock is responsible for most blunt trauma in our study. Sharp feathery edges were also found to cause penetrating injuries [17].

Players in the double game were found at higher risk of ocular injury than those involved in a single-player game [8,9,12]. On the contrary, we found that a higher number of ocular injuries occur in a single-player game, with the majority (83%) sustaining shuttlecock injuries from a smash shot by their opponents. A smash shot involves a high-velocity injury that causes severe coup and countercoup injuries to the eyes [9]. However, injuries due to badminton racquets are equally damaging [9,12]. Badminton racquet has been observed to cause enough force to knock someone down, break their spectacles and even cause rupture of the globe. However, no ocular injury due to the badminton racquet was documented in the present clinical audit.

The impact of blunt trauma or closed-globe injury is popularly described as the 'seven rings of trauma,' explaining the injuries' pathogenesis [18]. In this study, the reported ocular injuries involved all the 'seven rings of trauma.' The commonest was the presence of hyphaema. Microscopic and gross hyphaema is due to the shearing of the intraocular blood vessels, especially at the iris, during the compression (coup) and decompression (countercoup) mechanism in closed-globe injury [19]. Most are associated with angle recession in which there is a separation of longitudinal and circular muscle of the ciliary body. Other injuries to the iris, such as sphincteric tears, iridodialysis, and cyclodialysis cleft, may also be responsible for hyphaema [19,20]. Angle recession was reported to develop at 20-71% post-closed-globe injury regardless of the cause [21,22]. It was reported even higher (94-100%) in the presence of hyphaema [23,24]. In this clinical audit, hyphaema was detected in 96% of the cases. However, only 17 eyes developed angle recession, confirmed by gonioscopic findings. Due to the retrospective nature of this study, underreporting is most likely.

Traumatic angle recession glaucoma (TARG) has yet to be well defined. There is still no distinctive margin between those who just developed elevated IOP and glaucomatous changes. In our present audit, out of 17 eyes, only four developed glaucomatous changes, and eight had elevated IOP after a median follow-up time of 12 months. Perhaps, if the follow-up is longer, may be the percentage will be higher. It is estimated that 10% post closed-globe injury developed TARG in 10 years [25].

Due to the high velocity of the impact, there were also reported injuries to the lens and posterior segment. There were four eyes with subluxated lenses, and all of them had cataract operations with intraocular lens (IOL) implantation. In the case of a subluxated lens, the cataract operation is complicated. The type of IOL implantation depends on the remnant posterior capsule's availability and pupil size [26]. Traumatic mydriasis is common, which may deem inadequate for the stability of anterior chamber IOL. Therefore, scleral-fixated IOL may be more appropriate in the young age group. Scleral-fixated IOL is expensive and commonly conducted as secondary surgery [27]. This will incur extra costs and multiple hospitalizations.

In this clinical audit, traumatic retinopathy is one of the common presenting signs or sequelae post badminton-related injuries. Commotio retinae or Berlin edema was seen in five eyes. Posterior vitreous detachment (PVD) and vitreous hemorrhage were also documented in the present study, which may delay the detection of traumatic retinopathy. Commotio retinae are usually transient, but there are reported cases with poor visual prognosis [12,28]. Three out of five eyes with commotio retinae have a final visual outcome of worse than 6/18. Perhaps, due to the high velocity, the disruption of the outer segment of the photoreceptor was more severe and caused functional damage. However, the visual outcome may also be affected by other concurrent injuries, such as the subluxated lens and TARG.

A retinal tear was documented in one eye with myopia. In susceptible eyes, especially without protective wear, more sinister sequelae may occur. There were five emmetropic eyes: four myopic and one hyperopic. Although most of our patients regained good vision, this may be just a short-term outcome, but the sequelae are longer. The usage of protective eyewear, even during recreational sports, should be emphasized. Most badminton players were amateur, but even professional players were not protected from ocular injuries [12,13]. Although, the prevalence is much lower compared to the amateur badminton players. Campaign to prevent this preventable monocular blindness should be intensified further. Monocular blindness is significant to young adult life, with the loss of job opportunities and quality of life.

## Conclusions

In conclusion, ocular injuries related to badminton are common and cause detrimental effects on the long-term visual outcome. Traumatic hyphaema and commotio retinae are the most common presenting signs related to poor visual outcomes. Therefore, protective eyewear and promoting awareness of badminton-related ocular injuries is important to prevent monocular blindness in young adults.
